# Assessment of health safety of pigs taking natural sorbents with feed

**DOI:** 10.1186/s12917-022-03563-3

**Published:** 2023-01-06

**Authors:** Łukasz Wlazło, Małgorzata Kwiecień, Hanna Bis-Wencel, Wojciech Łopuszyński, Grzegorz Buszewicz, Katarzyna Karpińska, Izabela Rodzyń, Martyna Kasela, Paweł Sobczak, Bożena Nowakowicz-Dębek

**Affiliations:** 1 Department of Animal Hygiene and Environmental Hazards, Faculty of Animal Sciences and Bioeconomy, University of Life Sciences in Lublin, Akademicka 13, 20-950 Lublin, Poland; 2 Institute of Animal Nutrition and Bromatology, Faculty of Animal Sciences and Bioeconomy, University of Life Sciences in Lublin, Akademicka 13, 20-950 Lublin, Poland; 3 Department of Pathological Anatomy, Faculty of Veterinary Medicine, University of Life Sciences in Lublin, Głęboka 30, 20-612 Lublin, Poland; 4 Department of Forensic Medicine, Medical University of Lublin, Jaczewskiego 8B, 20-090 Lublin, Poland; 5 Department of Pharmaceutical Microbiology, Faculty of Pharmacy, Medical University of Lublin, Chodźki 1, 20-093 Lublin, Poland; 6 Department of Food Engineering and Machines, Faculty of Production Engineering, University of Life Sciences in Lublin, Głęboka 28, 20-612 Lublin, Poland

**Keywords:** Bentonite, Zeolite, Nutrient digestibility, Pigs, Health

## Abstract

**Background:**

The study assessed the effect of smectites (bentonite and zeolite) used as natural sorbents in the diet of pigs on feed digestibility, health parameters, the severity of anatomo-histological changes in organs, and the accumulation of volatile pollutants in organs.

**Material and methods:**

The study was conducted using fattening pigs (crossbreds from multiple breeds) assigned to three groups – a control (C) and two experimental groups (A and B), with 240 pigs in each group (3 replicates × 80). The animals in group C received a standard complete diet, while groups A and B received diets with 1.5% composed smectite sorbents. The feed and faeces were analysed for content of dry matter, crude ash, crude protein, ether extract, and crude fibre. The content of P was determined using a Helios Alpha UV–VIS spectrophotometer. Whole blood was analysed for haematological parameters and serum for biochemical parameters. Tissue samples were collected for analysis of volatile substances and histological analysis. After slaughter, samples of the lungs, liver, kidneys and jejunum were collected for morphological evaluation, and samples of the perirenal fat, liver, kidneys, lungs and brain for headspace gas chromatography (GC) to determine the levels of volatile toxic substances.

**Results:**

A statistical increase in the digestibility of crude fibre and an increase in that of P were observed in both experimental groups (A and B) in comparison to the control. The whole blood and serum of the pigs from the control group had statistically significantly higher levels of creatinine, urea, and Mg and a higher WBC count compared to both experimental groups (A and B).

**Conclusions:**

The feed additives were not shown to have a negative effect on the health parameters analysed or on accumulation of pollutants in selected tissues. No significant effect on the digestibility of most nutrients was observed; only an increase in the digestibility of crude fibre and a decrease in P digestibility were noted in the experimental groups.

## Background

Mineral feed supplements for animals are most often used in the form of pellets or powdered natural minerals, based mainly on aluminosilicates (bentonite, zeolite, or kaolinite) in combination with activated carbon or a carbohydrate complex, and natural polymers for technological purposes. The main purpose of their use in animal feed is to effectively immobilize most mycotoxins. In addition, they can have anti-clumping and anticoagulant properties. Apart from absorption of mycotoxins, well-selected mineral supplements in animal diets improve animals’ health status, the microclimatic parameters of the building, and zootechnical conditions [1]. Another reason for using mineral supplements in animal feed is their natural capacity to reduce gaseous pollutants [2]. European Union climate policy requires the agricultural sector to reduce emissions of gaseous pollutants. For this purpose, sorbents based on aluminosilicates are used as mineral feed supplements to effectively reduce the release of gaseous pollutants from farms [3–5]. According to the report of the EFSA Panel on Additives and Products or Substances used in Animal Feed, aluminosilicates and other smectite mineral supplements used as feed additives are safe for all animal species, consumers, and the environment in amounts of up to 2% of a complete diet. The results presented in the report confirm that smectites have no genotoxic properties [6]. Struciński et al. (2011) have demonstrated that due to their origin (as sedimentary rocks) and ion exchange properties, natural minerals can accumulate excessive amounts of dioxins, furans and their derivatives. For this reason it is important to assess the safety of their use in animal diets [7]. Previous research indicates that mineral supplements have beneficial effects in animal diets. Bentonite and zeolite used as mineral supplements have exhibited anti-diarrhoeal and bacterio- and fungicidal effects [8]. In addition, they can favourably influence production outcomes and animal health [9]. Added to bedding, they improve the microclimate of animal housing owing to their drying properties and capacity to reduce the amount of gases released, thus favourably influencing animal welfare [10]. These additives are safe in the amount of 0.2–1% of a commercial diet for broilers or pigs and have no negative effect on production, even improving poultry productivity [11]. Previous research has shown their effects on the fatty acid profile of kidney fat [12]. Appropriately chosen, small amounts of the sorbents used are an important factor in the safety of their application and potential negative effects on tissues and cells [13].

Although the effects of some natural smectite feed additives used as sorbents of gaseous pollutants are known, the effects of mixtures of these substances on animal health and the safety of their use are not fully understood. Therefore the aim of the study was to assess the effect of two natural sorbents, bentonite and zeolite, in diets for fattening pigs on feed digestibility, selected health parameters, the histological structure of selected internal organs, and the severity of pathological changes and accumulation of volatile substances in these organs.

## Materials and methods

The mineral supplements were previously tested for content of dioxins, furans, and dioxin-like polychlorinated biphenyls (PCBs). The study was conducted using fattening pigs that were crosses of multiple breeds (Choice-Genetics breeding line), assigned at random to three groups (control and two experimental groups). The initial body weight of the animals was about 35 kg. Each group comprised 240 individuals, kept in three pens with 80 animals in each pen. The experimental groups were designated A and B, and the control group as C. The animals received a standard complete diet adjusted for age (grower and finisher). The animals from groups A and B received a diet with 1.5% composed smectite sorbents, while group C received a standard complete diet. The animals were fed ad libitum with unlimited access to drinking water. Veterinary care was provided by a veterinarian employed on the farm. The microclimatic conditions on the farm (temperature, relative humidity, and air flow), which were systematically monitored throughout the experiment, were in compliance with animal welfare requirements [2].

### Feed and faeces

Feed and faeces were sampled twice for analysis of chemical composition, i.e. at the start (following a 14-day adaptation period) and end of the experiment. Apparent faecal digestibility was determined by the indicator method using 3 g Cr_2_O_3_ per kg of feed as an external marker [14]. Faeces were sampled in the morning from six animals in each group and pooled in a container for three days, after which the samples were dried for determination of the rate of drying, ground, and subjected to chemical analysis. Samples for analysis were weighed out from each portion of thoroughly ground faeces, and the concentrations of nutrients and insoluble ash were determined [15].

The apparent digestibility (faecal digestibility) of the nutrients was calculated from the following equation: apparent digestibility of nutrients = 100 − (100 × content of indicator in feed/content of indicator in faeces × content of nutrients in faeces/content of nutrients in feed).

Previously prepared feed was sampled from six bags, with randomization of the sample. The diets (*n* = 3) and faeces samples were analysed using AOAC methods [2012] for the content of dry matter (Method 925.09), crude ash (Method 923.03), crude protein by Kjeldahl’s method (Method 920.87), ether extract by Soxhlet extraction (Method 920.39) and crude fibre (Method 962.09). Total P content was determined according to Polish standard PN-76/R-64781 using a Helios Alpha UV–VIS spectrophotometer (Spectronic Unicam, Leeds, UK). Some of the results obtained for the content of nutrients and minerals in the diets are presented in Ossowski et al. [2]. Faecal digestibility was calculated as a percentage (ATTD), as apparent total tract digestibility of organic matter (OM), crude protein (CP), crude fat (CFat), and crude ash (CA) in the pigs’ diet. All analyses were performed in triplicate. The nutritional value of the pig feed was in compliance with nutritional recommendations [16].

### Analysis of blood parameters

Midway through the experiment, blood samples (10 ml) were collected from pigs from each group with similar body weight (average weight from pen about 70 kg per pig). Blood was sampled in the morning before feeding, from each replicate pen in each group, for analysis of haematological and biochemical parameters. From each replicate group 3 samples were collected, i.e. 9 per experimental group. Blood samples were collected from the cranial vena cava into single-use Vacutest tubes (VacutestKimaSrl.) containing lithium heparin. Within three hours of collection, whole blood was analysed for haematological parameters with a Mindray BC 500 Vet analyser (China). Plasma for analysis of biochemical parameters was obtained by centrifugation of whole blood at 3,000 rpm (603 × g) for 15 min in a laboratory centrifuge (MPW-350R, MPW Medical Instruments, Warsaw, Poland) at 4 °C. Samples were stored at − 80 °C until biochemical analysis. The concentrations of glucose (GLU), total protein (TP), creatinine (CREA), urea (UREA), magnesium (Mg), iron (Fe), and phosphorus (P) in the blood plasma were determined by spectrophotometry using Cormay tests. Activity of alanine aminotransferase (ALT), aspartate aminotransferase (AST), and alkaline phosphatase (ALP) was determined by spectrophotometry using Cormay tests. All spectrophotometric analyses were performed using a Mindray BS-120 spectrophotometer. Class A, G, and M immunoglobulins were determined in the blood plasma using ELISA assays (BiokomELx 808, Poland).

### Histological tissue analysis

After the fattening period animals from each group were slaughtered, and samples of the lungs, liver, kidneys, and jejunum were taken. These samples were fixed for 24 h in 10% neutral buffered formalin and absolute alcohol, and then passed through increasing concentrations of alcohol solutions and xylene in a tissue processor (Leica TP-1050) and embedded in paraffin blocks. Histological Sects. 4 µm thick, prepared using a sledge microtome (Leica SR-200) and stained with haematoxylin and eosin, were used for morphological analysis under a light microscope. For the liver samples, histochemical staining was additionally performed for the presence of neutral fats. For these analyses, the sections were fixed in 10% neutral buffered formalin, sliced in a freezing microtome (Cryotome FSE, Thermo Scientific), and stained with Sudan IV according to Daddi [17]. For visualization of glycogen, microscope sections of the liver were fixed in absolute alcohol and stained by the PAS method according to McManus [18].

### Analysis of gas residues

Analysis of toxic volatile substances was based on a method by T. Tankin and J.C. Crockett Butler [‘Blood Alcohol Analysis by Static headspace with Dual FID/Megabore Capillary Columns’, Terry Rankin, Jessie Crockett Butler, Thermo Electron Corporation Application Note: 10,076, Milan, Italy].

#### Chemicals

Analytical standards: acetone, ethanol, methanol, isopropanol (Multi-Component Alcohol Mix-100, Cerilliant, USA), 1-propanol, 2-butanol, 2-pentanol, bezene, dodecane, ethyl acetate, ethylbenzene, m,p,o-xylene (Supelco, Germany), toluene (Sigma-Aldrich, USA). Deionized water (Millipore, USA).

#### Sample collection and storage

After slaughter, the following material was collected: blood (1 ml), perirenal fat, liver, kidneys, lungs and brain (all 1 g). Biological samples were stored at -20 °C until analysis (Nowakowicz-Dębek et al. [19]).

#### Sample preparation

Samples in the amount of 200 μl of blood or 200 mg of tissue and 200 μl of internal standard (0.1 g/l tert-butanol solution in water) were placed in 10 ml headspace glass vials, closely crimped using a cap with a PTFE-silicone seal, and analysed by the HS-GC-FID technique.

#### Instruments and chromatographic conditions

TRIPlus headspace autosampler (Thermo Electron, United States) coupled to a Trace GC Ultra (Thermo Electron, United States) gas chromatograph equipped with one split/splitless injector (working temp. 200 oC) and two FID detectors (working temp. 250 °C). Chromatographic separation was carried out on a dual column system. Two capillary columns (parallel connection using Y-splitter) were used, i.e. Restek Rtx BAC1 and Rtx BAC2 30 m, 0.53 mm ID, 3 and 2 micron film, respectively (Restek, Bellefonte, PA, USA), working at a gradient temperature: initially 40 °C for 2 min, and then increased (15 °C/min) to 150 °C and held for 2 min. The carrier gas was helium, 18 ml/min. TRIPlus headspace autosampler work conditions: sample incubation 75 °C for 8 min, syringe temp. 80 °C, injection volume 0.3 ml. The operation of the gas chromatograph and the HS autosampler were controlled by X’calibur software, which was also used for data treatment. The analytical procedure was validated using certified analytical standards of volatile hydrocarbons (%RSD, LOD, LOQ, linear fit).

### Statistical analysis

The results of the haematological and biochemical analyses for each group were presented as means M with standard deviation (SD), standard error of the mean (SEM), and p-value. Statistical differences between groups were determined by Tukey’s test for a significance level of < 0.05. The analysis was carried out using Statistica software ver. 12.0 (StatSoft S.A., Tulsa, OK, USA). Means between groups marked with different letters (a, b…) differ significantly at *p* < 0.05.

## Results

Nutrient digestibility is presented in Table 1. In groups A and B the digestibility of crude fibre increased significantly (*p* = 0.001) relative to group C, by 11% and 4%, respectively. In the case of P, digestibility was lower (*p* = 0.0001) in groups A and B than in group C (by 5% and 11.6%, respectively). The chemical composition of the feed given to the animals is presented in Ossowski et al. [2].

**Table 1:** Apparent total tract digestibility (ATTD), %

Item	Nutrient digestibility, %			SEM	*p*-value
	C	A	B		
Crude protein	72.87	75.8	72.86	0.374	0.975
Crude fat	70.3	72.39	70.53	0.292	0.869
Crude ash	18.08	19.92	19.46	0.219	0.335
Crude fibre	48.93c	54.46a	51.02b	0.703	0.0001
BAW	64.07	63.80	64.34	0.101	0.098
P, g/kg	31.83a	30.19b	28.05c	0.444	0.0001

*SD* Standard deviation, *SEM* Standard error of the mean, *p* Probability value

The plasma levels of creatinine and urea in the pigs in the control group were significantly higher than in the groups receiving supplements (Table 2). Among the mineral compounds tested in the plasma, the Mg concentration in the control group (2.40 g/dl) was statistically significantly higher than in experimental group A (2.00 g/dl), at *p* < 0.05, by 20%. On the other hand, the concentrations of Fe and P in the plasma of all groups were similar: Fe from 235.74 µg/dl (group B) to 266.84 µg/dl (group C) and P from 8.78 g/dl (group A) to 9.31 g/dl (group C). The iron level showed tendencies close to statistical significance (Table 3).

**Table 2:** Analysis of selected blood biochemical parameters (M ± SD)

Item	C	A	B	SEM	*p*-value
	M ± SD	M ± SD	M ± SD		
Glu	90.25 ± 10.17	87.73 ± 12.07	88.36 ± 10.00	1.754	0.869
ALAT	55.76 ± 10.85	50.07 ± 3.86	54.27 ± 7.68	1.229	0.150
ASAT	61.40 ± 21.40	53.17 ± 22.90	44.54 ± 7.58	3.099	0.119
ALP	194.13 ± 48.34	214.87 ± 44.79	199.21 ± 39.38	7.11685	0.478
CREA	1.11 ± 0.07a	0.90 ± 0.10b	0.92 ± 0.04b	0.018092	0.0001
UREA	44.85 ± 4.34a	32.93 ± 7.05b	39.37 ± 5.75bc	1.239155	0.0001
TP	6.61 ± 0.39	6.09 ± 1.05	6.22 ± 0.26	0.118576	0.262
Mg	2.40 ± 0.15a	2.00 ± 0.36b	2.16 ± 0.29ab	0.054568	0.016
Fe	266.84 ± 36.45	242.77 ± 26.15	235.74 ± 23.78	4.84885	0.059
P	9.31 ± 0.46	8.78 ± 1.18	8.92 ± 1.27	0.180961	0.553

*SD* Standard deviation, *SEM* Standard error of the mean, *p* Probability value

**Table 3:** Analysis of white blood cell parameters in pigs during the experiment (M ± SD)

Item	C	A	B	SEM	*p*-value
	M ± SD	M ± SD	M ± SD		
WBC, 10^9^ l^−1^	16.54 ± 1.94a	13.49 ± 2.80b	15.18 ± 2.63ab	0.434	0.021
LYM, 10^9^ l^−1^	8.69 ± 1.70a	6.98 ± 1.27b	8.30 ± 1.52a	0.251	0.011
MID, 10^9^ l^−1^	2.00 ± 0.33	1.83 ± 0.53	1.92 ± 0.36	0.066	0.634
GRAN, 10^9^ l^−1^	5.86 ± 0.83	4.68 ± 1.31	4.96 ± 1.26	0.196	0.073
LYM, %	52.21 ± 5.37	52.29 ± 4.27	54.86 ± 4.81	0.748	0.243
MID, %	12.04 ± 1.94	13.22 ± 2.32	12.34 ± 1.29	0.298	0.261
GRA, %	35.74 ± 5.69	34.49 ± 3.86	32.79 ± 5.15	0.755	0.322

*SD* Standard deviation, *SEM* Standard error of the mean, *p* Probability value

Analysis of the white blood cell parameters of the blood of pigs during intake of supplements showed a significantly higher (*p* = 0.021) WBC count in the control group (16.54 × 10^9^/l) than in group A (13.49 × 10^9^/l), at *p* < 0.05. A higher LYM count (8.69 × 10^9^/l) was also noted in the control group in comparison to experimental groups A (6.98 × 10^9^/l) and B (8.30 × 10^9^/l), by 20.7% and 4.5%, respectively (Table 3).

Analysis of the red blood cell parameters of the blood of pigs receiving sorbents showed no statistically significantly differences (Table 4). The results of the haematological analysis of the blood of pigs were consistent with reference values [20].

**Table 4:** Analysis of red blood cell parameters in pigs

Item	C	A	B	SEM	*p*-Value
	M ± SD	M ± SD	M ± SD		
RBC, 10^12^/l	7.21 ± 1.57	6.62 ± 0.29	6.70 ± 0.40	0.125	0.183
HCT, %	36.30 ± 8.96	32.94 ± 2.11	34.06 ± 1.90	0.710	0.210
HGB, g dl^−1^	12.48 ± 2.88	11.61 ± 0.54	11.79 ± 0.80	0.227	0.356
MCV, fl	50.26 ± 2.79	49.81 ± 3.02	50.90 ± 2.83	0.447	0.567
MCH, pg	17.31 ± 0.46	17.59 ± 0.66	17.63 ± 0.47	0.086	0.360
MCHC, g dl^−1^	34.53 ± 1.76	35.43 ± 1.87	34.76 ± 2.14	0.304	0.480
RDW, **%**	19.86 ± 0.91	19.65 ± 0.71	20.32 ± 0.97	0.139	0.096
RDWa, fl	33.69 ± 2.54	32.78 ± 2.10	34.53 ± 2.00	0.352	0.085
PLT, 10^9^ l^−1^	215.67 ± 88.96	246.00 ± 69.31	246.00 ± 69.31	11.420	0.558
MPV, fl	7.18 ± 0.32	7.21 ± 0.50	6.93 ± 0.31	0.065	0.143

*SD* Standard deviation, *SEM* Standard error of the mean, *p* Probability value

**Fig. 1:**
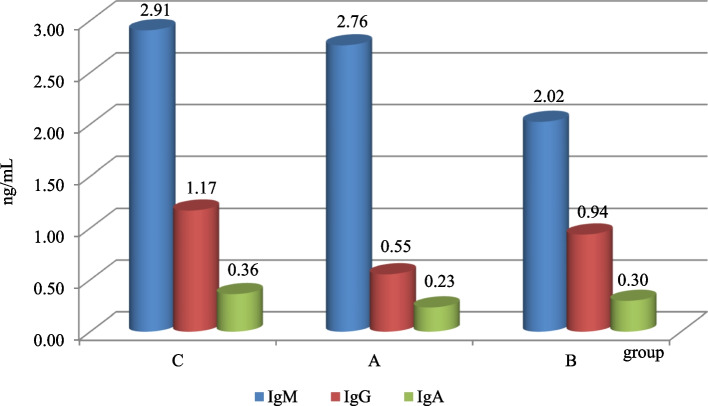
Immunoglobulin levels (ng/mL)

The level of class M immunoglobulins was in the range of 1.06–7.67 ng/ml (mean 2.91 ng/mL) in group C, 0.18–3.95 ng/ml in group A (mean 2.76 ng/mL), and 0.78–4.36 ng/ml in group B (mean 2.02 ng/mL). The average level of class G immunoglobulins was 1.17 ng/mL in group C, 0.55 ng/mL in group A, and 0.94 ng/mL in group B. The average IgA level ranged from 0.23 ng/mL in group A to 0.36 ng/mL in group B (Fig. 1).

**Fig. 2:**
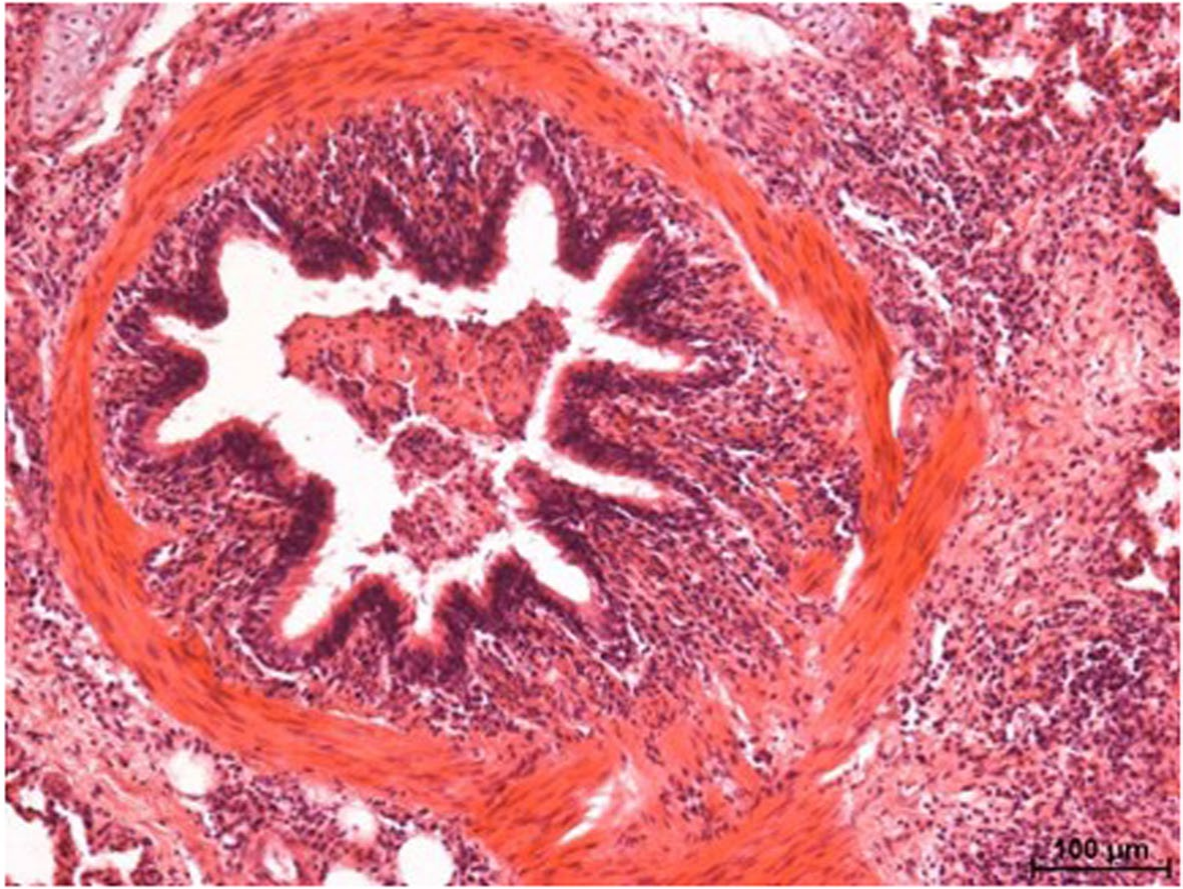
Exfoliated epithelial cells in the lumen of a bronchiole (control group). H & E staining. Approx. 100 × magnification

The microscope slides of the lungs showed multifocal moderate expansion of the alveolar septa, mainly in the vicinity of the small bronchi and bronchioles. The alveolar septa were infiltrated with inflammatory cells. The inflammatory infiltrates were mixed, including neutrophils, eosinophils and tissue macrophages, and in smaller numbers lymphocytes and plasmocytes. In the immediate vicinity of the small bronchioles, foci of proliferation of lymphoid tissue accompanied the bronchi. There were multiple foci of confluent areas of alveolar emphysema, characterized by thinning and gaps in the alveolar walls. Empty spaces were observed in the lung tissue, resulting from rupture of the alveoli, with bluntly terminated fragments of alveolar walls forming empty spaces. In the lumen of some of the bronchioles, exfoliated bronchiolar epithelial cells, necrotic masses, and excess mucous were observed (Fig. 2). The microscope image of the lungs of animals in the control (C) and experimental (A and B) groups did not differ significantly.

The microscopic examination of liver sections revealed no significant differences between the animals in the experimental and control groups. Glycogen was present in the form of fuchsinophilic granules unevenly filling the cytoplasm of hepatocytes. Granules were most abundant in the vicinity of the cell membrane; however, the inflammatory reaction was of uniform severity throughout the lobule. As in the case of the liver, no significant differences were observed in the microscope slides of the kidneys of animals between groups. The number of glomeruli appeared normal, with the Bowman’s space preserved, distributed evenly in the cortical layer, and various cross-sections of proximal and distal renal tubules lined with a cuboidal epithelium could be seen. Sudan IV staining did not reveal any deposits of lipid bodies within the tubular epithelium.

**Fig. 3:**
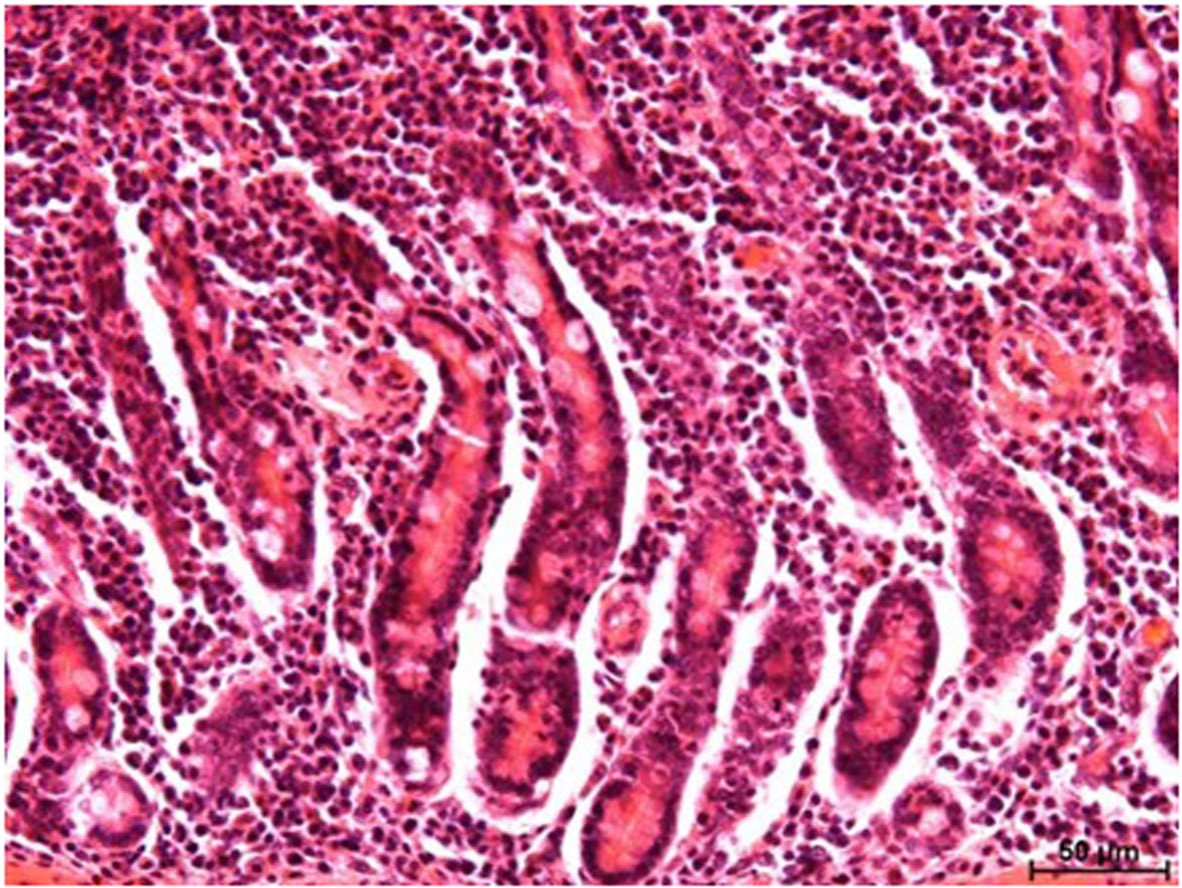
Lamina propria of the jejunal mucosa (control group). Visible inflammatory infiltrate consisting predominantly of mononuclear cells. H & E staining. Approx. 200 × magnification

Microscopic examination did not reveal significant differences in the appearance of the intestinal wall between groups of animals. Well-formed villi covered with a simple columnar epithelium could be seen, as well as cross-sections of intestinal crypts lined with a single-layered epithelium. A moderate number of mucous cells were visible between the enterocytes. In the experimental groups (A and B), there was an increased number of inflammatory cells within the lamina propria of the mucosa. The infiltrates consisted predominantly of lymphocytes and plasmocytes, with a smaller number of neutrophils and eosinophils (Fig. 3).

Neither acetone nor n-butanol was detected in the material (Table 5). The concentration of ethanol residues in the kidneys of the pigs was statistically significantly lower in group A (0.0495 mg/100 g) than in group C (0.0093 mg/100 g) at *p* < 0.05. The 2-propanol level was statistically significantly higher in the perirenal tissue of the animals from group C (0.418) than in groups A (0.068 mg/100 g) and B (0.104 mg/100 g), and the corresponding levels in the liver were C – 0.7768 mg/100 g, A – 0.1065 mg/100 g and B – 0.0205 mg/100 g. The highest concentration of n-propanol was obtained in the liver samples from group A, i.e. 0.0294 mg/100 g, which was statistically significant at *p* < 0.05. The presence of benzene was not detected in the kidney material from group C, while the amount of benzene in the samples from groups A (0.0265 mg/100 g) and B (0.0349 mg/100 g) were statistically significant. Pentanol was not detected in the perirenal fat of the group A animals, while the concentrations obtained in groups C (0.001 mg/100 g) and B were statistically significant (0.002 mg/100 g). The ethylbenzene concentration was statistically significantly higher in the blood (0.479 mg/100 g) and liver (4.4928 mg/100 g) of the pigs in group C. Dodecane was not detected in the perirenal fat of the pigs from group A, while in group C (0.008 mg/100 g) its level was statistically significantly higher than in group B (0.002 mg/100 g). The m-xylene concentration in the blood of the control group was statistically higher than in the experimental groups. In the samples of perirenal fat from group A there was significantly less m-xylene than in group B. The presence of o-xylene was not noted in the brain of animals from group B, and its concentration in group C was statistically significantly lower.

**Table 5:** Mean concentrations of toxic volatile substances (hydrocarbon derivatives) in the tissues of pigs (mg/100 g) (M ± SD)

Item		C	A	B	SEM	*p*-Value
		M ± SD	M ± SD	M ± SD		
Methanol	Blood	LOD	0.009 ± 0.005	LOD	0.002	0.076
	Perirenal fat	LOD	LOD	LOD	-	-
	Lungs	LOD	LOD	LOD	-	-
	Brain	LOD	0.009 ± 0.009	LOD	0.002	0.100
	Liver	0.002 ± 0.004	LOD	LOD	0.002	0.489
	Kidney	LOD	0.014 ± 0.004	0.008 ± 0.008	0.002	0.136
Ethanol	Blood	0.001 ± 0.002a	0.012 ± 0.0043b	LOD	0.002	0.0001
	Perirenal fat	0.240 ± 0.212	0.205 ± 0.074	0.129 ± 0.040	0.038	0.520
	Lungs	0.642 ± 0.782	0.762 ± 0.274	0.044 ± 0.042	0.1570	0.130
	Brain	0.028 ± 0.008	0.010 ± 0.005	0.360 ± 0.369	0.074	0.079
	Liver	0.099 ± 0.172	0.311 ± 0.137	0.112 ± 0.072	0.045	0.090
	Kidney	0.009 ± 0.002a	0.050 ± 0.033b	0.018 ± 0.010ab	0.007	0.020
2-Propanol	Blood	LOD	LOD	LOD	-	-
	Perirenal fat	0.418 ± 0.308a	0.068 ± 0.039b	0.104 ± 0.043bc	0.069	0.042
	Lungs	0.509 ± 0.463	0.305 ± 0.189	0.266 ± 0.113	0.0836	0.487
	Brain	0.005 ± 0.001	0.104 ± 0.029	0.232 ± 0.268	0.049	0.173
	Liver	0.777 ± 0.622a	0.107 ± 0.075	0.021 ± 0.041b	0.142	0.031
	Kidney	0.705 ± 0.083	0.410 ± 0.159	0.726 ± 0.323	0.064	0.066
n-Propanol	Blood	0.002 ± 0.0001	0.002 ± 0.001	0.001 ± 0.001	0.0001	0.133
	Perirenal fat	0.003 ± 0.0001	0.067 ± 0.039	0.104 ± 0.043	0.099	0.110
	Lungs	0.043 ± 0.048	0.027 ± 0.018	0.006 ± 0.003	0.0090	0.257
	Brain	0.010 ± 0.0001	0.001 ± 0.001	0.014 ± 0.014	0.003	0.130
	Liver	0.010 ± 0.010ab	0.029 ± 0.019a	0.003 ± 0.002b	0.004	0.029
	Kidney	0.005 ± 0.006	0.002 ± 0.002	0.010 ± 0.010	0.002	0.231
2-Butanol	Blood	LOD	LOD	LOD	-	-
	Perirenal fat	0.001 ± 0.001	0.013 ± 0.015	0.011 ± 0.013	0.003	0.219
	Lungs	0.001 ± 0.001	LOD	LOD	0.0001	0.125
	Brain	LOD	LOD	LOD	-	-
	Liver	LOD	LOD	LOD	-	-
	Kidney	LOD	0.002 ± 0.001	0.001 ± 0.001	0.0001	0.002
Toluene	Blood	LOD	LOD	LOD	-	-
	Perirenal fat	0.042 ± 0.072	LOD	LOD	0.0130	0.319
	Lungs	0.044 ± 0.088	LOD	LOD	0.044	0.405
	Brain	LOD	LOD	LOD	-	-
	Liver	0.109 ± 0.100	LOD	0.041 ± 0.083	0.024	0.154
	Kidney	LOD	LOD	LOD	-	-
Benzene	Blood	0.032 ± 0.023	0.042 ± 0.035	0.023 ± 0.029	0.009	0.633
	Perirenal fat	0.062 ± 0.047	0.023 ± 0.027	LOD	0.011	0.051
	Lungs	0.136 ± 0.154	0.083 ± 0.017	0.016 ± 0.018	0.0278	0.2203
	Brain	0.031 ± 0.007	0.016 ± 0.018	0.017 ± 0.019	0.005	0.439
	Liver	0.023 ± 0.028	0.086 ± 0.060	0.013 ± 0.026	0.014	0.051
	Kidney	LOD	0.027 ± 0.026a	0.035 ± 0.004b	0.005	0.010
Pentanol	Blood	0.001 ± 0.001	0.001 ± 0.001	LOD	0.0001	0.167
	Perirenal fat	0.001 ± 0.001a	LOD	0.002 ± 0.001b	0.0001	0.020
	Lungs	0.001 ± 0.002	LOD	LOD	0.001	0.130
	Brain	LOD	0.001 ± 0.001	0.001 ± 0.001	0.0001	0.269
	Liver	0.001 ± 0.001	0.002 ± 0.002	0.001 ± 0.001	0.0001	0.630
	Kidney	0.001 ± 0.001	0.001 ± 0.001	0.001 ± 0.001	0.0001	0.818
Ethylbenzene	Blood	0.479 ± 0.058a	0.329 ± 0.217ab	0.162 ± 0.138b	0.053	0.028
	Perirenal fat	2.486 ± 2.488	LOD	0.282 ± 0.326	0.520	0.075
	Lungs	11.110 ± 10.943	0.238 ± 0.275	0.247 ± 0.286	2.261	0.059
	Brain	LOD	0.278 ± 0.321	0.395 ± 0.457	0.098	0.257
	Liver	4.493 ± 2.670a	0.276 ± 0.319b	1.424 ± 2.511ab	0.765	0.039
	Kidney	4.470 ± 3.117	LOD	LOD	1.394	-
Dodecane	Blood	0.011 ± 0.010	0.002 ± 0.002	0.004 ± 0.001	0.002	0.099
	Perirenal fat	0.008 ± 0.007a	LOD	0.002 ± 0.003b	0.002	0.041
	Lungs	0.003 ± 0.005	0.007 ± 0.008	0.003 ± 0.003	0.002	0.512
	Brain	LOD	0.002 ± 0.003	0.004 ± 0.005	0.001	0.237
	Liver	0.005 ± 0.005	0.003 ± 0.003	0.003 ± 0.003	0.001	0.544
	Kidney	LOD	0.009 ± 0.009	0.005 ± 0.005	0.002	0.099
Ethyl acetate	Blood	0.001 ± 0.001	LOD	LOD	0.0001	0.033
	Perirenal fat	0.001 ± 0.001	0.002 ± 0.002	0.001 ± 0.0001	0.0001	0.225
	Lungs	0.001 ± 0.001	0.001 ± 0.0001	0.001 ± 0.001	0.0001	0.088
	Brain	LOD	LOD	0.0012 ± 0.0013	0.0003	0.1004
	Liver	0.001 ± 0.0001	0.001 ± 0.001	0.001 ± 0.001	0.0001	0.116
	Kidney	0.001 ± 0.0001	LOD	LOD	0.0001	-
m-Xylene	Blood	0.012 ± 0.008a	0.012 ± 0.007b	LOD	0.002	0.026
	Perirenal fat	LOD	0.005 ± 0.006a	0.016 ± 0.005b	0.002	0.0001
	Lungs	0.143 ± 0.272	LOD	0.007 ± 0.008	0.046	0.388
	Brain	0.007 ± 0.006	0.005 ± 0.006	0.009 ± 0.010	0.002	0.781
	Liver	0.150 ± 0.329	0.013 ± 0.001	LOD	0.057	0.504
	Kidney	LOD	0.022 ± 0.003	LOD	0.001	-
o- Xylene	Blood	0.005 ± 0.003	0.004 ± 0.003	0.0001 ± 0.0001	0.001	0.102
	Perirenal fat	0.006 ± 0.011	LOD	LOD	0.002	0.319
	Lungs	0.020 ± 0.018	0.006 ± 0.006	0.004 ± 0.005	0.004	0.151
	Brain	0.008 ± 0.0001a	0.011 ± 0.003b	LOD	0.002	0.0001
	Liver	0.053 ± 0.057	0.013 ± 0.015	LOD	0.011	0.129
	Kidney	LOD	0.007 ± 0.007	0.005 ± 0.004	0.001	0.110

Acetone and n-butanol were not identified in the material; LOD—below detection limit

## Discussion

Smectites are mineral supplements which are often used in feed. They can influence animal health, feed intake, and the palatability, digestibility, physical structure and even shelf-life of feed [21]. A basic marker used to assess animal health is changes in white and red blood cell parameters. In the present study, no significant changes were noted in the biochemical and haematological parameters of the blood. Similar conclusions arise from research by Holanda and Kim [22], who tested clay adsorbents in the diet of pigs and observed no deviations from physiological norms in blood parameters. A study of kaolin feed supplements for piglets conducted by Bederska-Lojewska and Pieszka [23] found no negative effect on blood parameters, while indicating a positive effect in the form of greater body weight gain than in the control group [23]. The values measured and the health markers used in the present study differed from those presented by other authors, but were within physiological ranges and did not differ significantly between group C and the experimental groups.

Environmental concerns are another argument in favour of the use of clay materials in the diet of pigs. Through their structure and sorption, used in the diet of pigs they enable retention of nitrogen and improve protein digestibility. No such effect was observed in the present study, but there was an increase in the digestibility of crude fibre and a decrease in the digestibility of P in the experimental groups in comparison with the control. The decrease in P digestibility in the experimental groups may have been due to a reduction in the enzymatic activity of phytase, which was immobilized on the sorbents. The enzymatic inhibition process seems to be the most likely explanation for the reduced P absorption from the diet [24]. This was confirmed in a study by Naik et al. [25], in which commercial products based on montmorillonite clay were used as phytase carriers. The authors observed 67% retention of the adsorbed enzyme [25]. Due to their very high absorption capacity, aluminosilicates may potentially influence the water and mineral balance of the body of pigs by binding important nutrients and macro- and microelements. This was not confirmed in the present study, in which only the plasma of pigs from the control group had statistically significantly higher levels of creatinine and urea than the groups receiving additives. Horky et al. ( 2022) also suggest that aluminosilicates are able to absorb many organic and inorganic substances in animals’ digestive tracts, but they do not affect mineral metabolism [26].

Macro- and microelements contained in smectites, especially kaolins, are rich in Fe compounds and can be utilized by animals to prevent mineral deficiencies and anaemia. This mechanism is confirmed by the phenomenon of geophagia, which has been observed as a natural method of correcting low Fe levels in some wild animals and in experiments on rats [27]. The biochemical analyses in the present study showed no increase in the Fe concentration in the plasma of pigs, with similar results obtained in all groups.

None of the differences in the experimental groups exceeded reference values, from which it can be concluded that the body compensates for the potential negative effect of mineral additives in the diet. Contrasting results were obtained by Zhang et al. [28] in an in vitro experiment which showed that bentonite negatively affects animal health through genotoxic activity, due to the size of the quartz grains contained in the dust. Alexopoulos et al. [29] observed that long-term use of clinoptilolite (2%) in the diet of pigs affected their biochemical and haematological parameters. As in the present study, the authors found no significant changes in haematocrit, leukocyte count or haemoglobin concentration (*p* < 0.05), but noted lower concentrations of urea nitrogen and cholesterol. In order to diversify the origin of the markers of health status used to evaluate the effects of natural mineral materials in the diet of pigs, the authors of the present study used several groups of parameters indicating potential differences in health status. Analysis of protein parameters of the immune system revealed no statistically significant differences indicating an immune imbalance in the animals. A study by Weaver et al. [30] showed that the addition of clay minerals and natural clays to the diet of pigs, through their protective effect against toxins, influences the immune system, stabilizing the number of monocytes and levels of immunoglobulins. This is confirmed by data presented by Zhang et al. [13], who report that bentonite in in vitro conditions can protect against cell damage. Microscopic examination showed no significant differences in the appearance and structure of the alveoli, intestinal wall, or liver tissue between groups of animals. The increased number of inflammatory cells in the lamina propria of the intestinal mucosa may be linked to the type of feed used, as an effect of hypersensitivity. Due to the content of silica, which is confirmed to have cytotoxic properties and to induce oxidative stress, additives based on aluminosilicates should be used in the lowest safe dose. Although changes in both the lungs and the intestines were visible at the microscopic level, their severity and extent indicate that the effect on the animals’ overall condition was minor. The liver and kidney samples showed no changes indicative of damage to these organs or indications that fat and carbohydrate metabolism differed between groups of animals. Similar results were presented by Trckova et al. [31] and Lee et al. [32]. The authors reported minor anatomo-histopathological changes in animals and no significant negative or positive effect on the histopathological assessment of the examined tissues. No significant microscopic changes in the organs or in the physiological structure of the tissues were shown in relation to the control group. Information presented by Horky et al. [26] is also in agreement with the present study as regards the lack of negative or toxic effect of bentonite clays in the diet of fattening pigs on tissues and the histological structure of the liver [26]. Poor housing conditions, like feed contamination, can affect the quality of animal products, in which residues of pollutants accumulate. Product quality can be affected by ions of some metals, halogenated hydrocarbons, and volatile compounds, especially fat-soluble compounds [19, 33]. The harmful effects of gaseous compounds released in animal production also apply to trace compounds, including propyl and butyl alcohols, whose high mobility in the body can cause them to accumulate in tissues and lead to kidney and liver damage. A study by Nowakowicz-Dębek et al. [19] confirmed that in animals exposed to air pollution, inflammatory histopathological changes in the liver and kidneys were intensified, and residues of these substances accumulated in the tissues and organs [34]. The differences observed in the amount of accumulated pollutants may be dictated by changes in the rate of metabolism of xenobiotics, the exposure time, and species-specific affinity for adipose tissue [35]. In the present study, residues of volatile toxic substances – hydrocarbon derivatives – were noted in the perirenal fat and in the liver, kidney, lung and brain tissue. However, the concentrations of the compounds between the experimental groups and the control group showed no statistically significant differences.

## Conclusions

The values measured and health markers used showed no significant differences between the control and experimental groups. The results presented suggest that sorbents used as feed additives are a safe component that does not reduce the digestibility of basic nutrients.

Well-selected and thoroughly tested aluminosilicates can be a promising feed material in the diet of pigs due to their ability to absorb gaseous pollutants in animal production.

## Data Availability

Most of the data generated or analysed during this study are included in this published article. The rest of data is available from the corresponding author on reasonable request.
